# Reciprocal inhibition and slow calcium decay in perigeniculate interneurons explain changes of spontaneous firing of thalamic cells caused by cortical inactivation

**DOI:** 10.1007/s10827-012-0430-8

**Published:** 2012-11-13

**Authors:** Jacek Rogala, Wioletta J. Waleszczyk, Szymon Łęski, Andrzej Wróbel, Daniel K. Wójcik

**Affiliations:** Department of Neurophysiology, Nencki Institute of Experimental Biology, 3 Pasteur St, 02-093 Warsaw, Poland

**Keywords:** Lateral geniculate nucleus, Perigeniculate nucleus, Cortical feedback, Calcium, Modeling

## Abstract

The role of cortical feedback in the thalamocortical processing loop has been extensively investigated over the last decades. With an exception of several cases, these searches focused on the cortical feedback exerted onto thalamo-cortical relay (TC) cells of the dorsal lateral geniculate nucleus (LGN). In a previous, physiological study, we showed in the cat visual system that cessation of cortical input, despite decrease of spontaneous activity of TC cells, increased spontaneous firing of their recurrent inhibitory interneurons located in the perigeniculate nucleus (PGN). To identify mechanisms underlying such functional changes we conducted a modeling study in NEURON on several networks of point neurons with varied model parameters, such as membrane properties, synaptic weights and axonal delays. We considered six network topologies of the retino-geniculo-cortical system. All models were robust against changes of axonal delays except for the delay between the LGN feed-forward interneuron and the TC cell. The best representation of physiological results was obtained with models containing reciprocally connected PGN cells driven by the cortex and with relatively slow decay of intracellular calcium. This strongly indicates that the thalamic reticular nucleus plays an essential role in the cortical influence over thalamo-cortical relay cells while the thalamic feed-forward interneurons are not essential in this process. Further, we suggest that the dependence of the activity of PGN cells on the rate of calcium removal can be one of the key factors determining individual cell response to elimination of cortical input.

**Electronic supplementary material:**

The online version of this article (doi:10.1007/s10827-012-0430-8) contains supplementary material, which is available to authorized users.

## Introduction

The thalamo-cortical loop and the role of cortical feedback in thalamic signal processing have been subject of extensive experimental studies and theoretical considerations over the last few decades (Kalil and Chase 1970; Baker and Malpeli 1977; Ahlsen et al. 1985; Deschenes and Hu 1990; Lindström and Wróbel 1990; Funke and Eysel 1992; Destexhe et al. 1998; Wörgötter et al. 1998, 2002; Bal et al. 2000; Einevoll and Plesser 2002). Most of the experimental investigations were concerned with facilitatory effects exerted by the cortex over thalamo-cortical relay (TC) cells. Experiments on cats revealed a decrease of spontaneous activity of TC cells as a result of inactivation of cortical input (Kalil and Chase 1970; Eysel et al. 1986; Wörgötter et al. 1998, 2002; Waleszczyk et al. 2005; Labra et al. 2006) while studies on monkeys showed no statistically significant changes in TC activity (Baker and Malpeli 1977; Przybyszewski et al. 2000).

The simplest view of the cortical feedback is excitation of TC neurons, so its suppression should lead to decrease of firing of lateral geniculate nucleus (LGN) neurons. This view is consistent with the observed decrease of firing rate of cat’s LGN cells caused by the suppression of cortical feedback (by cooling Wörgötter et al. 1998; Waleszczyk et al. 2005; TMS, Labra et al. 2006; or pharmacological application; Wörgötter et al. 1998). However, this hypothesis was largely based on observations of LGN cells activity only. Cessation of cortical input should decrease the activity of, targeted in parallel, thalamic interneurons, both of feed-forward (intrageniculate) and recurrent (located in perigeniculate nucleus, PGN – a part of cat’s thalamic reticular nucleus) types. The disinhibitory role of these disynaptic inputs on TC cells activity was postulated solely on the bases of the known circuitry of the network. Clearly, the interpretation of the role of each constituent of the system could be more reliable if the analysis involved simultaneous recordings from more cell classes.

In our experiments (Waleszczyk et al. 2005) we found that the cessation of cortical input decreased spontaneous activity of most of the LGN cells while at the same time it increased spontaneous activity of the majority of PGN interneurons. Thus, the experimental results seemed to be in agreement with the hypothesis of disinhibition of PGN cells, as suggested earlier by some authors (Wörgötter et al. 2002). The exact processes shaping the dynamics of the activity within thalamic circuits after cessation of cortical input remained, however, unclear.

This article aims to identify the putative mechanisms governing changes of activity observed in thalamic cells after cortical inactivation based on earlier experiments and suggested network topologies with special reference to our previous work (Waleszczyk et al. 2005) as one of the few studies investigating the effect of the cortex inactivation on both excitatory and inhibitory cells.

## Methods

To identify the basic mechanisms underlying functional changes in the thalamo-cortical loops following cortical inactivation we conducted a modeling study in NEURON simulation environment (Hines and Carnevale 1997), testing several network models with topologies suggested by earlier experimental and computational experiments. We constructed six different network models of conductance-based point neurons with rich ion channel repertoire capable of reproducing burst and tonic modes. A fundamental requirement was that the activity patterns of the model cells in simulated current clamp experiments should reflect those observed in physiological experiments. Our aim was to find a minimal model able to reproduce the experimental results of Waleszczyk et al. (2005) so each population was represented by a single neuron. This approach enabled us a closer study of the influence of different parameters of the model on its behavior in a reduced parameter space. We have also studied a large-scale network (>5000 neurons) variant of the most complex model VI obtaining results consistent with those obtained in the small network. However, the multitude of parameters in large models makes it difficult to unambiguously identify possible factors determining experimentally observed behavior.

### Details of simulation methods

Each network consisted of up to five cell types identified in anatomical and physiological studies (Ahlsen et al. 1985; Sherman and Guillery 1996):

- thalamo-cortical relay (TC) cell,
- recurrent inhibitory interneuron (PGN cell),
- LGN feed-forward interneuron (Int cell),
- cortical (Cx) cell simulating input to and output from the cortex (layers 4 and 6),
- The retinal (Ret) input to the thalamus was provided in the form of gamma spike trains, following the observations of Troy and Robson (1992).

The simulated thalamic cells were based on previous physiological and modeling investigations (Huguenard and McCormick 1992; McCormick and Huguenard 1992; Destexhe et al. 1994; Pape and McCormick 1995; Zhu and Uhlrich 1997; Zhu et al. 1999; Halnes et al. 2011) and included all identified currents (listed below in sections describing specific Cell models). All parameters of thalamic model cells were adjusted to fit their responses to injected currents to those observed in the physiological experiments mentioned above. The network topologies were based on experimental data and computational research dealing with cortical feedback effects on thalamic activity (Ahlsen et al. 1985; Wörgötter et al. 1998; Debay et al. 2001; Hillenbrand and van Hemmen 2001; Einevoll and Plesser 2002; Waleszczyk et al. 2005).

All the model networks were driven with spike trains simulating spontaneous retinal input. Following the results of Troy and Robson (1992) we assumed the inter-spike intervals were independently and identically distributed with the gamma distribution $f(X)={{\left( {{\kappa \left/ {\mu } \right.}} \right)}^{\kappa }}{X^{{\kappa -1}}}{{{\exp \left( {-{{{\kappa X}} \left/ {\mu } \right.}} \right)}} \left/ {{\varGamma \left( \kappa \right)}} \right.}$ of order *κ* = 5 and mean rate of 1/*μ*. (We have also performed additional simulations with homogeneous Poissonian input and found qualitatively the same results – not shown). Available literature data on the firing rate of cat retinal ganglion cells mostly refers to the activity evoked by visual stimuli and quotes rates in the range of 36–120 Hz (Castelo-Branco et al. 1998; Kara et al. 2000). Passaglia et al. (2001) quotes the firing rate of 50 Hz in response to steady uniform full-field illumination, which could be interpreted as spontaneous activity. Kara et al. (2000) showed that the retinal activity is about twice higher than the firing rate of LGN cells. Given spontaneous LGN activity within the range of 3–40 Hz (Sherman and Guillery 2001; Waleszczyk et al. 2005) this gives spontaneous retinal activity within the range of 6–80 Hz. Taking into account the above considerations we assumed 50 Hz as the default retinal input frequency for the purpose of our investigations.

In every sequence of simulations we took the same realization of noisy retinal input to simplify comparison of results before and after elimination of cortical input and between various models.

The firing rate of cells – the only measure reported in all the experiments with cortex inactivation (Kalil and Chase 1970; Eysel et al. 1986; Wörgötter et al. 1998, 2002; Waleszczyk et al. 2005; Labra et al. 2006) – was used to compare our results to those from the earlier experiments and between the studied models. The metric used was the average firing rate over 30 runs of 1 s blocks per each experimental setup. To assure trial independence necessary for *t*-test comparisons, before performing t-tests we checked each pair of compared runs (TC with cortex input versus TC without cortex input and PGN with cortex input versus PGN without cortex input) with chi-square test of independence.

Each simulation sequence consisted of thirty runs of 1 s. Models consistent with experimental results for 50 Hz retinal inputs were also tested with the retinal input signal of 10, 30, 50, 70 and 90 Hz to test their properties in the admissible frequency range of spontaneously firing retinal ganglion cells, which we found very strongly affecting the frequency of the TC cells.

Wherever applicable we compared simulation results of both TC and PGN cells to data obtained in our original physiological experiments (Waleszczyk et al. 2005). Results were compared using the two-tailed *t*-test and were said to be statistically significant for *p* < 0.05. All simulations were run with NEURON 7.1 simulation software. Spikes were counted within one-second runs with spike threshold of 10 mV.

### The network – description of model cells, their connections and network topologies

All network topologies, cell models and synaptic connections we used were based on data taken from published material (either physiological results or theoretical models). As our goal was to identify and test the simplest possible model still reproducing experimental data, we used the same synaptic reversal potentials and time constants for a given synapse type (excitatory or inhibitory) throughout the networks. Using the same model cells and the same framework of point neuron networks for all models allowed direct comparison of the generated results. We did not implement any form of plasticity in our models.

Estimating parameter values of neural models is usually a challenge since the model dynamics can be sensitive to small changes of the parameters. Moreover, many of them are difficult to estimate due to necessity of scaling down the network in terms of model cells complexity, the number of cells and connections. Thus, the parameters, such as the numbers of cells in modeled assemblies and the numbers of connections encountered in experimental conditions, must be adjusted often basing mainly on personal experience and judgment (Destexhe et al. 1998). In our simulations the cell conductances and synaptic weights initially derived from literature were extensively tested and adjusted to fit experimental results.

### Cell models

We used single-compartment conductance-based models with the membrane potential changing according to ${C_m}\frac{dV }{dt }={I_{inj }}-\sum\limits_j {I_j }$ including all the ionic currents *I*_*j*_ identified in a given cell type (listed below in sections describing specific Cell models). All the parameters were manually tested and adjusted with current injections to model cells placed outside the network with no incoming or outgoing connections (which we call in the following *dissociated cells*) to fit experimental *in vitro* activity of neurons (Huguenard and McCormick 1992; McCormick and Huguenard 1992; Pape and McCormick 1995; Zhu and Uhlrich 1997; Zhu et al. 1999) or the known modeling results following experimental data (Destexhe et al. 1994; Halnes et al. 2011).

#### Thalamo-cortical relay cell

The TC model cell included currents I_T_, I_A_, I_K2_ and I_H_ identified by Huguenard and McCormick (1992), and I_Na_, I_C_, I_L_, fast I_Na_ and I_K_, and calcium dynamics described by McCormick and Huguenard (1992). Summarized conductance data for model TC cell are presented in Table 1, full equations for all the currents of all the cells are given in the Appendix.

**Table 1:** Maximum conductivities of channels (S/cm^2^) and other membrane parameters used in the models of different cells

Parameters	Description	TC	PGN	Cx	INT
C_m_ (uF/cm^2^)	membrane capacity	0.29	1	1	1
I_K,max_ (S/cm^2^)	Fast K+ current	0.044	0.01	0.036	0.01
I_na,max_ (S/cm^2^)	Fast Na+ current	0.09	0.1	0.12	0.16
I_Nap,max_ (S/cm^2^)	Persistent K+ current	7.99e-04			
I_T,max_ (S/cm^2^)	Voltage dependent low threshold Ca2+ current	7.95e-03	7e-03		5.2e-3
I_Lmax_ (S/cm^2^)	High threshold Ca2+ current	6e-03			5e-4
I_K[Ca],max_ (S/cm^2^)	Ca2+ dependent K+ current		0.06		0.06
I_C,max_ (S/cm^2^)	Ca2+ activated K+ current	1e-07			
I_CAN.max_ (S/cm^2^)	Ca2+-dependent nonspecific cation current		2.5e-4		2.5e-4
I_K2,max_ (S/cm^2^)	Slowly inactivating K+	5e-03			
I_H,max_ (S/cm^2^)	hyperpolarization-activated cation current	1.4e-05			2e-03
I_A,max_ (S/cm^2^)	Rapidly inactivating potassium current	2.4e-3			
I_Naleak,max_ (S/cm^2^)	Sodium leak current	5e-06			
I_Kleak,max_ (S/cm^2^)	Potassium leak current	7e-07			
I_leak,max_ (S/cm^2^)	Non-specific leak current		5e-11	3e-4	6e-08
Calcium dynamics					
Depth [μm]	depth of the shell beneath the membrane	0.1	1		0.1

**Fig. 1:**
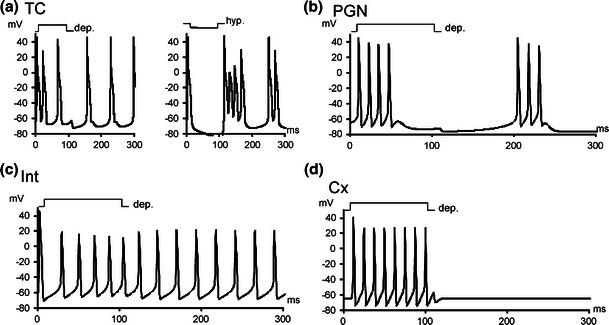
Activity of the dissociated model cells (outside of the network, stimulated by current injection) used in the study (**a**) tonic mode of TC cell in response to depolarization and hyperpolarization currents (**b**) bursting activity of PGN cell in response to hyperpolarization current (PGN model cell did not exhibit tonic activity) (**c**) activity of feed-forward interneuron (Int) in response to depolarizing current (**d**) activity of cortical (Cx) cell in response to depolarizing current

As shown in *in vivo* and *in vitro* experiments TC cells can exhibit two modes of activity – tonic spiking at the cell depolarization and burst firing at hyperpolarization (Huguenard and McCormick 1992; Sherman and Guillery 1996). Likewise, the TC model cell used in simulation also reproduced both modes of activity (Fig. 1(a)). The model TC cell was tuned to reproduce the above modes with firing rate within 10–30 Hz (Sherman and Guillery 1996; Kara et al. 2000; Waleszczyk et al. 2005; Andolina et al. 2007).

#### Perigeniculate cell (PGN cell, recurrent inhibitory interneuron)

PGN model cell included currents I_T_, I_CAN_, I_KCa_, I_K_, I_Na_, and calcium dynamics, and was tuned to exhibit intrinsic bursting following Destexhe et al. (1994). A summary of conductances used is given in Table 1. An example of activity of the PGN model cell is shown in Fig. 1(b). Despite tuning the PGN cell so that it was bursting when isolated, within a network it could function in either tonic or bursting mode, similarly to what was observed in the rat and the cat (Leresche et al. 1991; Contreras et al. 1993; Waleszczyk et al. 2005). Membrane mechanisms used in the PGN model cell were taken from the ModelDB database (Accession No. 3343, originally developed by Alain Destexhe). The PGN cell was tuned to fire within the range of 8–15 Hz (Waleszczyk et al. 2005).

#### Feed-forward LGN interneuron

There are only few studies on electrical properties of feed-forward LGN interneurons. The currents used in the model of feed-forward interneuron, I_T_, I_CAN_, I_KCa_, I_K_, I_Na_, I_L_ and I_H_, were identified by Zhu et al. (1999) in the rat thalamus, and recently also found by Halnes et al. (2011) in the mouse LGN. We used the appropriate currents from TC and PGN model cells and adjusted parameters to fit to physiological responses of feed-forward interneurons observed by Pape and McCormick (1995). The results of modeling of Int activity are presented in Fig. 1(c).

The reported firing rate of feed-forward LGN interneuron ranges from 7–15 Hz (Pape and McCormick 1995) to 10–20 Hz (Lorincz et al. 2009). Pape and McCormick (1995) also noticed the higher firing rate of the feed-forward LGN interneuron than TC cells. Having tuned TC cell firing rate within the range of 10–30 Hz we decided to keep the feed-forward LGN interneuron firing within a range of 15–40 Hz.

#### Cortical cell

Since we were not interested in cortical processing but in the effects of turning off the cortical influence on thalamic cells we used the Hodgkin-Huxley model (Hodgkin and Huxley 1952) as a proxy for a Cx cell. The results of modeling of Cx activity are presented in Fig. 1(d).

### Synapses

In all the network models we used only GABA_A_ and AMPA receptors. Since the essential response characteristics of thalamic cells seem not to depend on the properties of NMDA receptors (Debay et al. 2001; Hillenbrand and van Hemmen 2001, 2002) we decided not to include these receptors in the networks. Additionally, we ruled out GABA_B_ receptors from the modeled networks since most of the GABAergic inhibition in the cat’s lateral geniculate nucleus seems to be acting via GABA_A_ receptors (Sherman and Koch 1986; Lindström and Wróbel 2011). Another reason to exclude GABA_B_ from our investigations was to keep the models reproducing experimental results as simple as possible, even though GABA_B_ receptors may play a role in the PGN–TC network (Crunelli and Leresche 1991; Mistry et al. 2008).

Synaptic currents were adapted from the thalamocortical model by Traub et al. (2005) (Accession No 45539). In particular, the AMPA synapse was modeled as the alpha function with the decay time constant set to 0.8 ms and the reversal potential of 0 mV. The GABA_A_ synapse was modeled as exponential decay with the reversal potential of −80 mV and the decay time constant of 10 ms.

### Network topology

In our simulations we considered topologies consistent with currently known anatomy and physiology of the mammalian visual system (Ahlsen et al. 1985) and, for comparison, those used in earlier investigations. All models were restricted to specific connections within the retino-geniculo-cortical system (at the cortical level only the first order projection areas were taken into account); all the other incoming and outgoing connections (e.g. neuromodulatory) were neglected. The networks were tuned to attain experimentally observed spontaneous firing rates expressed by most of the investigated TC cell (range 5–40 Hz, Sherman and Guillery 2001; Waleszczyk et al. 2005), and PGN cells (range 5–40 Hz) (Waleszczyk et al. 2005). Our goal was to identify which topological and dynamical aspects of the network were crucial for the observed experimental effects of cortical inactivation (Waleszczyk et al. 2005).

#### Model I

**Fig. 2:**
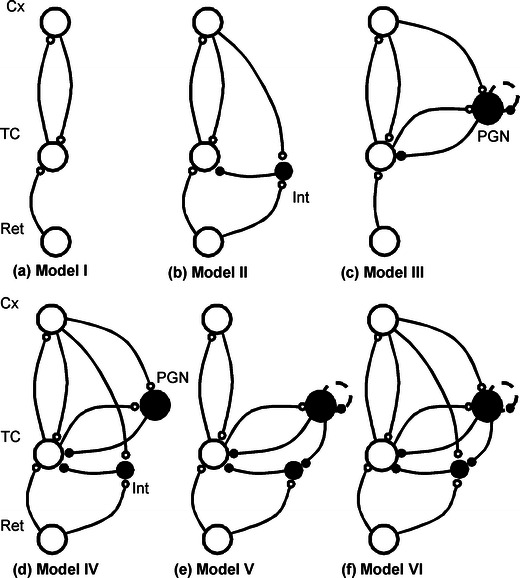
Network topologies of the studied models of cortico-thalamic circuitry (**a**) Model I: Simple thalamo-cortical loop (**b**) Model II by Wörgötter et al. (1998) (**c**) Model III by Debay et al. (2001) (**d**) Model IV by Einevoll and Plesser (2002) (**e**) Model V by Hillenbrand and van Hemmen (2001) (**f**) Model VI by Ahlsen et al. (1985). *Large circles* – cells, *small circles* – synapses. *White symbols* – excitatory elements, *black symbols* inhibitory elements. *Broken line* – reciprocal inhibition between PGN cells, Ret – retinal ganglion cell, LGN – principal LGN cell, Int – feed-forward interneuron, PGN – recurrent interneuron, Cx – cortical cell

The simplest model (Fig. 2(a)) consisted of only two reciprocally connected neurons, with the TC neuron driven by retinal input. This maximally reduced model was used only to test a hypothesis whether the inactivation of cortical feedback is sufficient to explain the observed decrease of the firing rate in TC cells when other elements of thalamo-cortical loops are neglected.

#### Model II

The second model implemented the scheme used by Wörgötter et al. (1998) who investigated modulation of temporal responses of TC cells of the cat exerted by cortical inactivation (cooling and application of GABA) or activation (application of glutamate) and interpreted experimental results within the context of Model II shown in Fig. 2(b). Model II is Model I expanded with the LGN feed-forward interneuron (Int) receiving cortical excitatory input.

#### Model III

The third model includes a representation of PGN interneuron population with reciprocal connections. There are physiological (Bal et al. 1995; Sanchez-Vives et al. 1997), morphological (Uhlrich et al. 1991; Deleuze and Huguenard 2006) and modeling (Bal et al. 2000; Debay et al. 2001) indications for such connections. We tested two representations of such a scheme: (i) two mutually inhibiting PGN cells, and (ii) one PGN cell with self-inhibition through an autosynapse, and the results were equivalent (Model III; Fig. 2(c)). In the following (Models V and VI) we decided to use the latter representation. Note that the Model III provides also a good representation of the thalamo-cortical loop of the rat somatosensory system whose specific recurrent inhibitory interneurons are placed in the thalamic reticular nucleus (Debay et al. 2001; Huguenard and McCormick 2007).

#### Model IV

This model was equipped with the feed-forward Int and recurrent PGN interneurons without reciprocal connections (Fig. 2(d)). Such a scheme was originally proposed by Dubin and Cleland (1977) and studied later by Sherman and Koch (1986).

#### Model V

This network was based on an extensive study of thalamic input to cortical cells conducted by Hillenbrand and van Hemmen (2001, 2002) who utilized a simplified thalamo-cortical computational model (Fig. 2(e)). This model lacked cortical input to PGN and feed-forward LGN interneurons, but included reciprocal connections between PGN cells and connections between the recurrent and feed-forward interneurons.

#### Model VI

The most complex model contained all the cells and connections described above including inhibitory connections from recurrent (PGN) to feed-forward LGN interneurons (Int) (Ahlsen et al. 1985); (Fig. 2(f)).

Connections between the cells in different models are summarized in Table 2

**Table 2:** Connections between model cells in the investigated topologies. X – marks an existing connection

	Model II	Model III	Model IV	Model V	Model VI
Ret-TC	X	X	X	X	X
Ret-Int	X		X	X	**X**
TC-Cx	X	X	X	X	**X**
TC-PGN		X	X	X	**X**
Int-TC	X		X	X	X
PGN-PGN		X		X	X
PGN-TC		X	X	X	X
PGN-Int				X	X
Cx-TC	X	X	X	X	X
CX-Int	X		X		X
Cx-PGN		X	X		X

.

### Axonal delays

It is well-known that axonal delays play a major role in oscillatory activity of neuronal networks and affect their stability (Crook et al. 1997; Ermentrout and Kopell 1998; Foss and Milton 2000; Dahlem et al. 2009; Brandstetter et al. 2010). To find out the influence of delays on the activity of the cortico-thalamic system we tested various delay times of the direct inhibitory and excitatory connections to TC, PGN and Int cells on TC cell firing rate. The initial synaptic delay values we used (Table 3) were taken from the papers by Wörgötter et al. (1998) and Traub et al. (2005).

**Table 3:** Axonal delays (ms) between different model cells used in tested networks

Axonal delays (ms)	Ret →	TCR →	PGN →	Int →	Cx →
→ Ret					
→ TCR	1		1	1	5
→ PGN		1	1		1
→ Int	1	1	1		5
→ Cx		1			

### Weights of synaptic connections

Weights of synaptic connections within a network are among the most difficult parameters to set in a modeling study due to the incompleteness of experimental data. Typically, they are tuned by varying the weights of individual synapses, the number of synapses (Adams and Cox 2002), or they are set arbitrarily (Gollo et al. 2009) depending on implemented model, available data or tested hypothesis. In all models investigated in our work the circuitry was based on anatomical and physiological data available for studied connections.

### Network tuning and testing

Parameters of the cells, after adjustment, were frozen and tuning of the network was limited to modifying synaptic weights. We required the network behavior after cortex inactivation to be consistent with the experiment (decrease of the TC cells activity and increase of the PGN cells activity). The only cell allowed to increase its firing rate after cortex inactivation was PGN as observed in the physiological experiment (Waleszczyk et al. 2005). Allowed firing rate of all model cells was set in the range of 5–40 Hz.

We adopted a three-step tuning and testing procedure: (i) setting the initial parameter range by manual tuning, (ii) systematic search in the parameter space and (iii) stability testing.

(i) Manual tuning
  We decided to set the initial parameters for model VI (Fig. 2(f)), which included all anatomically or experimentally identified connections in the cat thalamus (Dubin and Cleland 1977; Ahlsen et al. 1985), and which contained all the other investigated models as subsets. Initial absolute synaptic weights were set to 0.5 for all connections and adjusted in consecutive iterations till the model fit the experimental results (Kalil and Chase 1970; Funke and Eysel 1992; Wörgötter et al. 1998, 2002; Hillenbrand and van Hemmen 2001, 2002; Waleszczyk et al. 2005). Thus obtained values were used as indicators for setting parameter ranges for further steps.
(ii) Systematic search in the parameter space
  Having found one set of synaptic weights compatible with the experimental data we performed a systematic search in the parameter space around that particular set to make sure that the compatibility with experiment is retained under changes of parameter values, at least in some neighborhood.
  Because of the high dimensionality of the problem it was not feasible to use a grid-search approach, that is to choose a number of values for each parameter and test all combinations (if we chose just four values of each parameter we would have to test more than 4 million combinations). Instead, we chose randomly *N* = 50.000 samples with each parameter taken from a uniform distribution over the tested range. For each of the samples we run a pair of simulations with the cortex being active or inactive, and tested whether such combination of parameters led to a model compatible with the experiments (which we call a positive sample) or not (a negative sample).
  Specifically, we marked a sample as positive if and only if all of the following conditions were satisfied:
  1. the firing rate (averaged over 30 trials) of the TC, PGN and Int cells was in the range from 5 to 40 Hz,
  2. the firing rate of the TC and Int cells decreased, and the firing rate of the PGN cell increased after cortex inactivation,
  3. the differences listed in 2) were statistically significant (*t*-test, *p* < 0.05).
    To obtain the other Models (II to V) appropriate connections were removed from Model VI and the simulations were repeated with the same parameters as for Model VI. Only the cell types present in each model were tested for compliance with conditions 1)-3) listed above, that is the tests for the PGN cell were skipped in Model II and the tests for the Int cell were skipped in model III. Existence of positive samples was treated as an indicator whether a given model topology was consistent with the experimental results.
(iii) Stability testing
  As the final step we tested whether the positive samples form a ‘stability island’ in the space of parameters, that is, a region such that for points deep inside it small perturbations of the parameters preserve the compatibility with experimental constraints. The existence of a stability island is necessary for the model to be biologically plausible.
  First we propose a candidate parameter set for a model. This can be simply a mean value of parameters over all positive samples. However, we found that a better (more stable) set can in some cases be proposed by employing a statistical classifier (Support Vector Machine with a Gaussian kernel) trained on the available positive and negative samples (we used the Python package scikits.learn described in Pedregosa et al. 2011). Such a classifier provides a decision function F of the parameters which, within the accuracy of the classifier, returns values greater than zero on the positive parameter sets, and smaller than zero for negative samples. One can find the maximum of F, which should correspond to a parameter set far from the boundary and well within the stability island (‘optimal’ parameters). We used a modified objective function with an additional “sum of squares” term which pushed the solution towards the middle of the parameter range. However, the details of how a candidate parameter set is obtained (whether as a mean of positive samples or through more sophisticated methods) are not crucial, as we further test the stability of that set, that is, we test whether the model remains compatible with the experiment under changes of parameters.
  The stability of the chosen parameter sets was tested in two ways. First, a grid search was performed on a two-dimensional subspace spanned by two of the parameters (synaptic weight between TC and PGN cells, and the weight of the self-inhibition of the PGN cell).
  Second, we run a number of simulations for the randomly chosen parameter values close to the ‘optimal’ ones, allowing all 11 parameters to change at the same time, and used the results of the simulations to quantify the stability of the ‘optimal’ parameter set.

## Results

### Different network topologies result in different activity changes after cortical inactivation

#### Model I

The simplest explanation for the decrease of TC cell activity after inactivation of cortical areas is the removal of cortical feedback to LGN. To test this possibility we used the simplest model which contained only TC and Cx cells driven with retinal input. We first took absolute synaptic weights from Model VI. Since in Model I the only inputs to TC cell were from the retina and the cortex, their relative ratio, after elimination of the remaining connections, was higher than typically used in more complete networks. To obtain more realistic system we tried different ratios of incoming retinal/cortical TC inputs keeping constant their cumulative total weight and then relaxing this constraint. In all cases inactivation of the cortical cell resulted in *increased* activity of TC neuron.

#### Other models

For models II to VI we performed a systematic search in the parameter space in the manually found parameter ranges (shown in Table 4), as described in the Methods.

**Table 4:** Synaptic weight ranges obtained for Model VI via manual tuning

Synaptic strengths	Ret →	TCR →	PGN →	Int →	Cx →
→ Ret					
→ TCR	0.01–0.2		0.01–1	0.001–0.2	0.1–2
→ PGN		0.1–2	0.01–1		0.2–4
→ Int	0.001–0.02		0.01–0.2		0.01–0.2
→ Cx		0.01–2			

Only the models III and VI (models including reciprocally connected PGN cells driven by cortical input) produced positive samples (that is models which gave firing rates within the assumed range and led to decreased activity of the TC cell and increase in PGN activity after cortex inactivation) in significant numbers (19.575 out of 50.000 for model III, 6.755 for model VI; models II, IV, and V produced no positive samples). We have repeated the search for models II, IV, and V using broader parameter ranges (upper limits increased five times compared to presented in Table 4) to make sure we did not miss a region of positive solutions. We have not found positive samples in the broader ranges either. Note that the parameter scan for model VI was done in a space of larger dimension than for model III, so a direct comparison of the ‘size of the parameter space’ for the two models is not justified.

These results support the conclusion that it is not possible to choose synaptic weights in models II, IV and V in a way compatible with experimental results, at least not in the studied broad range of parameters. Therefore, we excluded them from further studies.

Model VI included all anatomically or experimentally identified cell types and connections in the cat’s visual thalamus, while model III, also reproducing experimental results, represents the known cat’s anatomy less well, which is why we chose Model VI as the most adequate representation of the studied part of the system.

The systematic search in the space of synaptic weights we describe above was performed for a single, fixed set of membrane parameters obtained through manual tuning. It is possible, however, that a different set of parameters might lead to consistence with electrophysiological experiment (Waleszczyk et al. 2005) in topologies we excluded. To test this hypothesis we performed two additional tests for Models IV and V. First, for each of these two topologies we found a single set of synaptic weights (from among the 50.000 used in the systematic search) which resulted in possibly smallest violation of the network activity constraints listed above in the “Network tuning and testing” section. We then fixed the synaptic weights and varied the membrane parameters for all the model cells (within the range of +/− 50 %), testing 50.000 different random combinations. Second, we varied both the synaptic weights and the membrane parameters simultaneously (we paired each of the 50.000 sets of synaptic weights with one of the 50.000 sets of membrane parameters).

These two tests yielded numerous (~10.000) positive samples for Model IV and a small number (~100) for Model V. However, we found out that while the network activity in these positive samples was plausible, the individual cells (TC, PGN and INT) failed to reproduce physiological behavior in current clamp simulations identical with the simulations performed while fitting the membrane parameters of the model cells. Specifically, for each positive sample at least one of the three cells (TC, PGN or Int) exhibited undesired behavior for either depolarizing or hyperpolarizing current (not shown). These test results explain that the positive results of network simulations found for Model IV and V were possible only when individual cell models were pushed beyond the limits of proper physiological behavior.

### Stability with respect to the synaptic weights

Having established Model VI as the model of interest we further checked its stability. A large number of positive samples (~10 % in case of model VI) suggests the existence of a stability island in the space of parameters, and indeed such a region can be found. As stated in the Methods, we begin the stability analysis by choosing a candidate parameter set. The analysis of the full 11-dimensional space using a statistical classifier yielded an ‘optimal’ set of parameters given in Table 5. Next, we analyzed the stability of that parameter set.

**Table 5:** Values of synaptic strengths for the representative model obtained in a scan over the space of models

Synaptic strengths	Ret →	TCR →	PGN →	Int →	Cx →
→ TCR	0.128		0.732	0.0365	0.926
→ PGN		0.701	0.255		3.11
→ Int	0.00871		0.143		0.116
→ Cx		0.107			

**Fig. 3:**
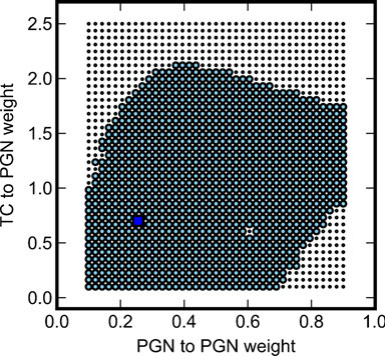
Stability of Model VI in two-dimensional space where the weights of connections from PGN to PGN and from TC to PGN are changed. *Small dots* denote negative samples, *larger circles* – positive samples (consistent with experiment). The *square* marks the parameters values shown in Table 5

In Fig. 3 we show the stability analysis in two dimensions. One can see that the chosen ‘optimal’ parameter set lies well within the region of positive solutions.

We further tested the stability of that solution in the full 11-dimensional space. We chose randomly a number (*N* = 50.000) of parameter sets, with a constraint than no single parameter is changed by more than 50 %. We simulated Model VI for these parameter sets and found that only a small fraction (171 out of 50.000, less than 0.35 %) were negative samples. Moreover, the bulk of the negative samples were located quite far from the ‘optimal’ solution: for 146 negative samples (>85 %) the sum of the percent changes of the parameters was greater than 100 %.

One could state that the relatively small Ret–Int and Int–TC connection weights in the set of ‘optimal’ parameters, bring the model closer to Model III, but the sensitivity of Model VI to delays between Int and TC suggests high influence of the Int cell on TC activity in spite of the small weight (see the Section Stability of network activities against changes in connection delays). Moreover, it is also possible to find good, stable solutions while keeping these two weights fixed at higher values, e.g. equal to 0.01 and 0.1 respectively (not shown).

To test the influence of a richer spectrum of synaptic receptors on the systems studied we repeated our numerical experiments expanding the models with NMDA and GABA_B_ synapses. The ratios of NMDA to AMPA and GABA_B_ to GABA_A_ were set constant to values reported by many publications (Young and Chu 1990; Yang et al. 2003; Watt et al. 2004). In particular, the ratio of NMDA:AMPA was set to 1:4, while the ratio of GABA_B_: GABA_A_ was set depending on a connection, since most of the GABA_B_ receptors are probably located in reticular thalamic nuclei (Destexhe et al. 1994; Bowery et al. 1999). For PGN-PGN connections the ratio of GABA_B_: GABA_A_ was set to 1:4 (Young and Chu 1990; Yang et al. 2003) and 1:20 for the other connections. The simulation results for the modified models where consistent with those with no GABA_B_ and NMDA synapses.

### Results for different input frequencies

We also tested the behavior of Model VI within supposed range of spontaneous retinal activity 10–90 Hz (see the Methods), specifically we tested responses of the TC and PGN cells to cortex inactivation in the following retinal input frequencies: 10, 30, 50, 70 and 90 Hz.

**Fig. 4:**
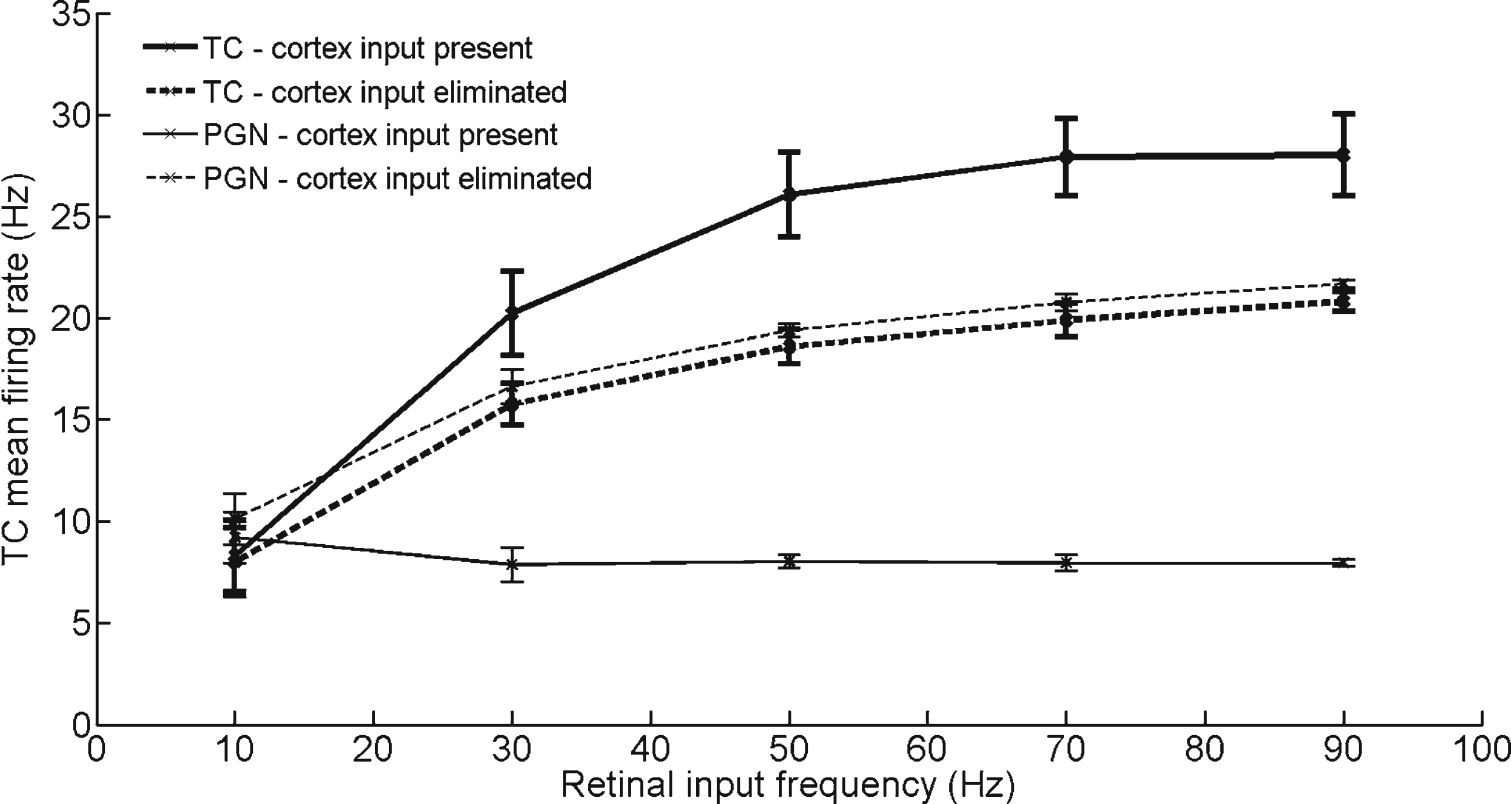
Firing rates of TC and PGN cells calculated for Model VI with different retinal input frequencies. The differences in firing rates between states with active and inactive cortex were significant at input frequencies over 10 Hz (*p* > 0.01; one tailed *t*-test)

The results are shown in Fig. 4. Only at the lowest retinal input frequency (10 Hz) the elimination of cortical input did not change firing rates significantly, neither in TC nor in PGN. At all higher retinal input frequencies the elimination of cortical input evoked significant firing rate changes for PGN and TC cells (*P* < 0.01).

### Putative biophysical mechanisms which could influence experimentally observed changes of thalamic cells activity

**Fig. 5:**
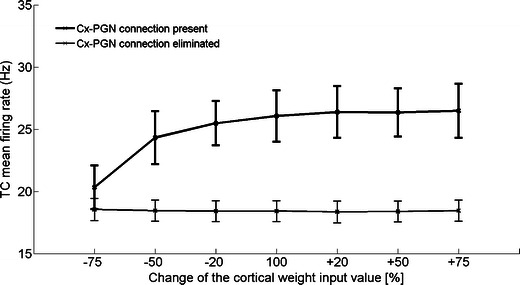
The effect of elimination of Cx–PGN connection on TC firing rate in Model VI. Elimination of cortical input to PGN makes TC cells insensitive to changes of cortical input. Synaptic weights from the cortex in Model VI and its variant with eliminated Cx–PGN connection were reduced by 20, 50 and 75 % or increased by 20, 50, 75 and 100 % with respect to the original settings

All models consistent with experimental results were characterized by a presence of cortical drive to PGN. To investigate the role of this connection in normal activity we studied the activity of TC cells in Model VI and its variant with eliminated Cx–PGN connection with rescaled weights from the cortex. Cortical weights were reduced by 20, 50 and 75 % or increased by 20, 50 and 75 % with respect to the original settings. TC firing rate of the original Model VI was sensitive to changes of cortico-thalamic input weights, although not all differences were significant, while in the variant with eliminated Cx–PGN connection, TC firing rate was constant regardless of weights’ changes (Fig. 5).

**Fig. 6:**
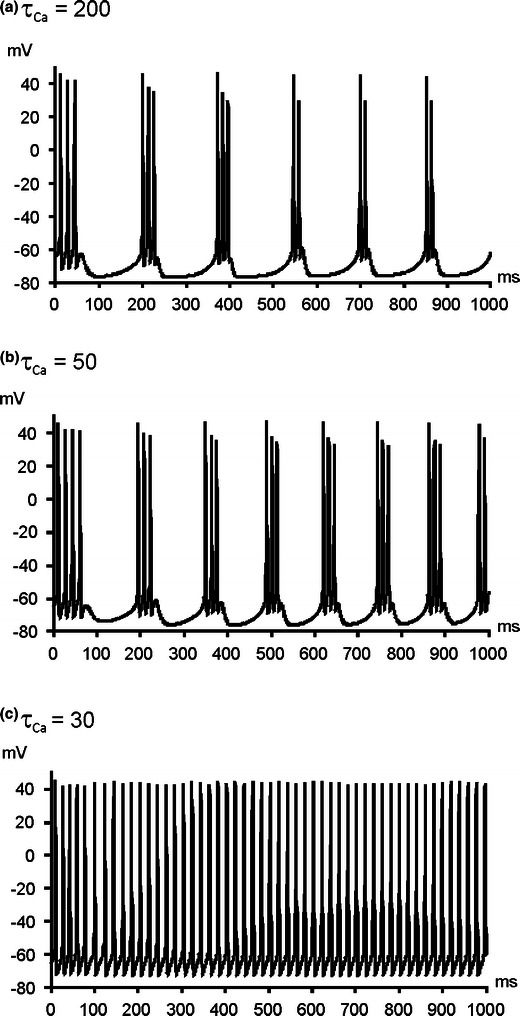
Dependence of firing rate of depolarized, dissociated PGN model cell on the time constant of calcium removal rate (τ_Ca_). Decrease of the time constant from 200 ms to 50 ms increased the number of spikes in the bursts, shortened interburst intervals, and finally led to persistent firing, when set at 30 ms

To identify possible biophysical mechanisms behind specific changes of activity of TC and PGN cells we investigated possible factors in dissociated model cells. Both types of cells may exhibit two different modes of activity – tonic and bursting (see Methods). We started with PGN cells which exhibit stronger tendency for bursting and therefore seem to be more susceptible to changes in factors influencing its mode of activity. Following the experimental results of Sugaya et al. (1988) who found a relation between bursting activity and calcium concentration changes we decided to test the properties of our PGN model cell with different calcium removal rates. The reported ranges of the calcium removal time constant τ_Ca_ vary from 1–16 ms (Aradi and Holmes 1999) to 20–800 ms (Majewska et al. 2000). Reducing τ_Ca_ from 200 to 30 ms in dissociated (out of the network) PGN model cell changed its firing mode from bursting to fast tonic spiking (Fig. 6).

We chose the range 1–800 ms for τ_Ca_ in the PGN cell to check sensitivity of the Model VI to changes in calcium kinetics.

**Fig. 7:**
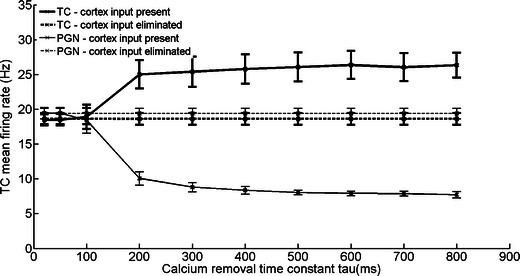
The effect of calcium removal rate on firing frequency of TC and PGN cells in Model VI. The slower calcium removal in PGN cell the higher the difference between firing frequencies of PGN and TC cells before and after elimination of cortical input. Retinal input: 50 Hz

After placing the PGN cell back in the network its activity was mostly tonic and its firing frequency slowly decreased with increasing τ_Ca._ Slower calcium removal (large τ_Ca_) led to higher differences between the firing rates of PGN and TC cells (Fig. 7). For fast calcium removal (low τ_Ca_), changes in the firing rate were negligible. TC and PGN cells with similar firing rates (5–30 Hz for TC and 5–35 Hz for PGN cells) were recorded in our physiological experiment (Waleszczyk et al. 2005).

Changes of calcium removal rate in TC model cells did not lead to significant changes of TC and PGN firing rates in the reference Model VI.

### Stability of network activities against changes in connection delays

The effects of delays of direct and indirect connections to TC cell on its firing rate were tested using different delay values. Initial delays were set to 1 ms for all connections but cortico-thalamic which were set to 5 ms (Tsumoto et al. 1978; Traub et al. 2005). The sensitivity of firing rates to various delays was tested by changing the initial delay by 2 ms up and down where possible, at input frequencies of 10, 30, 50, 70 and 90 Hz. Stability of resulting rates against changes of delay values in all synapses was tested separately keeping all other parameters unchanged. In all investigated input frequencies Model VI was robust against most changes of delays between involved neurons Table 6 shows tested delay values and their significance to changes of TC cell firing rate.

**Table 6:**
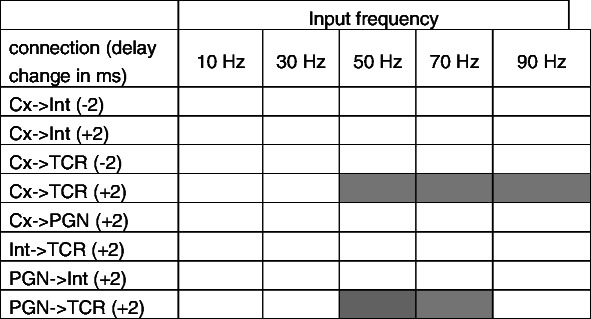
Sensitivity of model VI to changes in axonal delays at different retinal input frequencies. Axonal delays in the connections indicated in the first column were changed by +/− 2 ms. Statistically important changes with respect to the initial settings are indicated by shaded fields

## Discussion

We have investigated six classes of point-neuron models of thalamo-cortical loops, with each class representing a different network topology. The models were constrained by requiring consistency of simulated current clamp activity patterns of individual cells with electrophysiological experiment (Waleszczyk et al. 2005), and by the ratio of the number of connections incoming to the thalamus. The main findings are:

- a simple network of reciprocally connected TC and cortical neurons (Model I) cannot reproduce experimentally observed changes of TC cell activity after inactivation of the cortex;
- to reproduce experimentally observed changes of activity following cortical inactivation, i.e. decrease of TC cell and increase of PGN interneuron firing, the network should contain PGN interneurons with mutul inhibition and a cortico-perigeniculate connection (Models III and VI);
- dependence of the activity of PGN cells on the calcium removal rate (calcium concentration) could play an important role in determining individual cell response to elimination of cortical input.

In Model I we observed increased activity of TC cells after inactivation of cortex throughout all investigated input frequencies and weight settings.

We were not able to fit Models IV and V to the experimental data within imposed physiological constrains on the parameters, therefore, we disqualified these models as candidates for explaining experimentally observed changes of spontaneous activity within cortico-thalamic system. It should be noted, however, that relaxing constraints imposed on three main cell types (TC, PGN, Int; requiring all of them reproducing correct activity patterns observed in physiological current clamp experiments) could give positive samples in Model IV, but not Model V.

Overall, our results show that only models with PGN driven by both TC and Cx, with mutual inhibition between PGN cells – that is Model III and Model VI – could fully reproduce available experimental results. This indicates that the strong cortical innervation of PGN and reciprocal PGN connections (Huguenard and McCormick 2007) are necessary for tuning TC sensitivity to cortical drive. Yet the evidence for reciprocal PGN inhibitory connections is not very strong. Many physiological and morphological experiments were conducted on juvenile mice (Landisman et al. 2002; Deleuze and Huguenard 2006) however Warren and Jones (1997) showed that RTN cells response are changing their pattern up to P21. Morphological investigations on adult cats show presence of collaterals within PGN in majority of cells enabling reciprocal connections (Uhlrich et al. 1991).

Thus, we support the hypothesis that thalamo-cortico-perigeniculate loop serves as a regulatory feedback tuning sensitivity of thalamus to cortical excitation as postulated previously by Lindström and Wróbel (1990, 2011) and Waleszczyk et al. (2005).

We conclude that Model VI containing feed-forward interneuron and reciprocally connected PGN inhibitory interneurons driven by cortical input, originally proposed by Ahlsen et al. (1985), is the best representation of the studied part of the cat visual system, as it both well reproduced results from our physiological experiment (Waleszczyk et al. 2005) and earlier investigations, and includes all anatomically identified elements of the thalamo-cortical network. Interestingly, the results of our physiological experiments could be reproduced not only by Model VI, including all anatomical elements of the studied part of cat’s visual cortico-thalamic network, but also by Model III, which lacks the feed-forward LGN interneuron (Int). This indicates that feed-forward interneurons are not essential for tuning of TC cells to cortical drive. Indeed, for a long time they have been postulated to play a different role, namely in shaping center-surround interactions of TC cells’ receptive fields by increasing local contrast (see Lindström and Wróbel 2011 for review).

These results also support the conclusion mentioned in our previous papers (Lindström and Wróbel 1990, 2011; Waleszczyk et al. 2005) that the main role exerted by the modulatory cortico-thalamic pathway from layer 6 serves facilitation of the ascending retino-cortical flow of visual information at the level of LGN. Further, our findings strongly indicate an important role of mutual inhibition between the PGN cells. Most probably, the enhanced spontaneous activity in PGN neurons after cortical inactivation reflects a decrease of their mutual inhibition following a decrease of excitatory cortical input.

While tuning the models to experimental data we found that the parameter describing the rate of calcium release from geniculate cells specifically influenced network activity, probably through changes of ionic conductances (LeMasson et al. 1993; Siegel et al. 1994; Giugliano 2001) influencing the firing mode, tonic or bursting, of PGN and TC cells (Robinson et al. 1993; Williams and Stuart 1999; Nishimura et al. 1996). These findings support our conjecture that changes in the PGN activity following cortical inactivation (Waleszczyk et al. 2005) may be caused by changes in calcium concentration also affected by physiological, activity dependent cellular mechanisms. This may also indicate a more general role of the calcium as an important factor in cell responses to depolarization.

Experimental results obtained by Baker with Malpeli (1977) and Przybyszewski et al. (2000) call for a separate comment. In these papers the authors did not observe significant effects of V1 inactivation on spontaneous firing of TC cells which were observed in our experiment (Waleszczyk et al. 2005). The lack of changes in the spontaneous activity could be explained by simulation results obtained in this study at lower retinal input frequencies (Fig. 4) or lower calcium removal rate constants in PGN cell (Fig. 7). In all simulations where the above mentioned conditions were met, elimination of cortical input did not produce significant changes in TC activity. Which of these factors could cause the stabilization of spontaneous rate in these experiments (Baker and Malpeli 1977; Przybyszewski et al. 2000) requires further investigations.

As a final disclaimer, note that our results must be considered in the specific context they were derived, which is for networks of point neurons, including most identified membrane mechanisms, and with single neurons representing individual populations. It is not inconceivable that extending the scope of the model, for instance increasing the representation of each group, adding morphology and diversifying channel distribution, etc., we might obtain variants of models based on the remaining topologies (i.e. other than III or VI) consistent with available experimental data including our physiological experiments. On one hand, following this route would defeat the purpose of this work, which was obtaining of the simplest possible model still reproducing changes in thalamic neuronal activity following cortical inactivation. On the other hand, simulating larger networks based on the topologies studied here we have not been able to reproduce our experimental results unless the topology was that of model III or VI which is why we consider such a possibility highly improbable.

## Electronic supplementary material

Below is the link to the electronic supplementary material.

ESM 1 (PDF 150 kb)
