# Supplementary material for: Reciprocal inhibition and slow calcium decay in perigeniculate interneurons explain changes of spontaneous firing of thalamic cells caused by cortical inactivation
Source: J Comput Neurosci. 2012 Nov 13;34(3):461–76. doi: 10.1007/s10827-012-0430-8 (PMC3650241; doi:10.1007/s10827-012-0430-8)
Supplement: Supplementary file 1 — (PDF 150 kb) [file 10827_2012_430_MOESM1_ESM.pdf]

Appendix: Current equations used in cell models

Thalamo-cortical model cell (TC)

The TC model cell includes currents described in McCormick and Huguenard (1992) and Huguenard and McCormick (1992). For the convenience of the reader we gather all the equations here.

General equation describing the change in intracellular potential V in TC model cell:

$$C_m \frac{\mathrm{d}V}{\mathrm{d}t} = -I_{\mathrm{inj}} - I_{\mathrm{Na}} - I_{\mathrm{Nap}} - I_{\mathrm{L}} - I_{\mathrm{T}} - I_{\mathrm{C}} - I_{\mathrm{A}} - I_{\mathrm{K2}} - I_{\mathrm{h}} - I_{\mathrm{Kleak}} - I_{\mathrm{Naleak}}.$$

The membrane capacity C_m and the maximum conductivities $I_{\bullet,\max}$ are listed in Table 1.

Transient Na$^+$ current I_{Na}

The membrane current density I_{Na} is given by:

$$I_{\mathrm{Na}} = I_{\mathrm{Na,max}}(V - E_{\mathrm{Na}})m^3 h,$$

where $E_{\mathrm{Na}} = 50$mV is the equilibrium potential for Na$^+$, m is the activation variable and h is the inactivation variable. The steady-state m and h are described by

$$m_\infty = \frac{\alpha_m}{\alpha_m + \beta_m}, \quad h_\infty = \frac{\alpha_h}{\alpha_h + \beta_h},$$

and activation (inactivation) time constants are:

$$\tau_m = \frac{1}{\alpha_m + \beta_m}, \quad \tau_h = \frac{1}{\alpha_h + \beta_h}.$$

For the activation variable m of the I_{Na} current we take:

$$\alpha = 0.091(V + 38)\left[1 - \exp\left(-\frac{V + 38}{5}\right)\right]^{-1},$$

$$\beta = -0.062(V + 38)\left[1 - \exp\left(\frac{V + 38}{5}\right)\right]^{-1},$$

V measured in mV, whereas for the inactivation variable h we use:

$$\alpha = 0.016 \exp\left(\frac{-55 - V}{15}\right)$$

$$\beta = 2.07\left[\exp\left(\frac{17 - V}{21}\right) + 1\right]^{-1}.$$

Persistent Na$^+$ current I_{Nap}

The current density is given by:

$$I_{\mathrm{Nap}} = I_{\mathrm{Nap,max}}(V - E_{\mathrm{Na}})m,$$

where $E_{\mathrm{Na}} = 49$mV, and

$$m_{\mathrm{Nap}\infty} = \left[1 + \exp\left(\frac{-49 - V}{5}\right)\right]^{-1},$$

$$\tau_m = 1\mathrm{ms}.$$

Low threshold Ca^{2+} current I_L

For the I_{L} current the Goldman-Hodgkin-Katz equation is used:

$$I_{\mathrm{L}} = I_{\mathrm{L,max}} m^2 z^2 \frac{VF^2}{RT} \frac{[\mathrm{Ca}^{2+}]_{\mathrm{i}} - [\mathrm{Ca}^{2+}]_{\mathrm{o}} \exp(-\frac{zFV}{RT})}{1 - \exp\left(-\frac{zFV}{RT}\right)},$$

where $z = 2$ for Ca$^+$, F is the Faraday constant, R is the gas constant, T is the temperature and $[\mathrm{Ca}^{2+}]_{\mathrm{i}}$, $[\mathrm{Ca}^{2+}]_{\mathrm{o}}$ are intracellular and extracellular concentrations of Ca^{2+}. The gating variables α, β for the activation variable m are:

$$\alpha = 1.6\left\{1 + \exp\left[-0.072(V - 5)\right]\right\}^{-1},$$

$$\beta = 0.02(V - 1.31)\left[\exp\left(\frac{V - 1.31}{5.36}\right) - 1\right]^{-1}.$$

Voltage activated Ca^{2+} current I_T

For this current we use the same equation as for I_{L} above, but with the maximum conductivity $I_{\mathrm{T,max}}$ and with an activation-inactivation term $m^2 h$ in place of m^2.

Activation kinetics (m) for the I_{T} current:

$$\tau_m = \left[\exp\left(\frac{V + 132}{-16.7}\right) + \exp\left(\frac{V + 16.8}{18.2}\right)\right]^{-1} + 0.612.$$

Inactivation kinetics (h) for I_{T} current:

$$\tau_h = \begin{cases} \exp\left(\frac{V+467}{66.6}\right) & \text{if } V < -80\mathrm{mV}, \\ \exp\left(\frac{V+22}{-10.5}\right) + 28 & \text{if } V \geq -80\mathrm{mV}. \end{cases}$$

Activation variable asymptotic value m_∞:

$$m_\infty = \left[1 + \exp\left(\frac{V + 57}{-6.2}\right)\right]^{-1}.$$

Inactivation variable asymptotic value h_∞:

$$h_\infty = \left[1 + \exp\left(\frac{V + 81}{4}\right)\right]^{-1}.$$

Ca^{2+} activated K^+ current I_C

$I_{\mathrm{C}} = I_{\mathrm{C,max}}(V - E_K)m$

Gating variables for the I_{C} current:

$$\alpha = 2.5 \cdot 10^5 [\mathrm{Ca}^{2+}]_{\mathrm{i}} \exp\left(\frac{V}{24}\right),$$

$$\beta = 0.1 \exp\left(\frac{-V}{24}\right).$$

Rapidly inactivating K^+ current I_A

$I_{\mathrm{A}} = I_{\mathrm{A,max}}(V - E_{\mathrm{K}})m^4 h,$

Activation kinetics for the I_{A} current:

$$\tau_m = \left[\exp\left(\frac{V+35.8}{19.7}\right) + \exp\left(\frac{V+79.7}{-12.7}\right)\right]^{-1} + 0.37,$$

$$m_\infty = \left[1 + \exp\left(\frac{V+60}{-8.5}\right)\right]^{-1}.$$

Inactivation kinetics for the I_{A} current:

$$\tau_h = \begin{cases} \left[\exp\left(\frac{V+46}{5}\right) + \exp\left(\frac{V+238}{-37.5}\right)\right]^{-1} & \text{if } V < -63\text{mV}, \\ 19 & \text{if } V \geq -63\text{mV}, \end{cases}$$

$$h_\infty = \left[1 + \exp\left(\frac{V+78}{6}\right)\right]^{-1}.$$

Second inactivating K^+ current I_{K2}

$I_{\mathrm{K2}} = I_{\mathrm{K2,max}}(V - E_{\mathrm{K}})mh.$

Activation kinetics for the I_{K2} current

$$\tau_m = \left[\exp\left(\frac{V-81}{25.6}\right) + \exp\left(\frac{V+132}{-18}\right)\right]^{-1} + 9.9,$$

$$m_\infty = \left[1 + \exp\left(\frac{V+43}{-17}\right)\right]^{-1}.$$

Inactivation kinetics for the I_{K2} current

$$\tau_h = \left[\exp\left(\frac{V-1329}{200}\right) + \exp\left(\frac{V+130}{-7.1}\right)\right]^{-1} + 120,$$

$$h_\infty = \left[1 + \exp\left(\frac{V+58}{-10.6}\right)\right]^{-1}.$$

Voltage dependent I_h current

$I_{\mathrm{h}} = I_{\mathrm{h,max}}(V - E_{\mathrm{h}})m,$

Activation kinetics for I_{h} current:

$$\tau_m = \frac{1}{\exp(-14.59 - 0.086V) + \exp(-1.87 - 0.0701V)}$$

$$m_\infty = \left[1 + \exp\left(\frac{-(V+49)}{5}\right)\right]^{-1}$$

Sodium leak current I_{Naleak}

$I_{\mathrm{Naleak}} = I_{\mathrm{Naleak,max}}(V - E_{\mathrm{Na}})$

Potassium leak current I_{Kleak}

$I_{\mathrm{Kleak}} = I_{\mathrm{Kleak,max}}(V - E_{\mathrm{K}})$

Ca^{2+} buffering

The change in the intracellular calcium concentration $[\mathrm{Ca}^{2+}]_{\mathrm{i}}$ due to diffusion is calculated according to:

$$\frac{\mathrm{d}[\mathrm{Ca}^{2+}]_{\mathrm{i}}}{\mathrm{d}t} = 1.0 \cdot [\mathrm{Ca}^{2+}]_{\mathrm{i}}$$

where time is in ms. The concentration of Ca^{2+} in time step t was calculated according to:

$$[\mathrm{Ca}^{2+}]_{\mathrm{i},t} = [\mathrm{Ca}^{2+}]_{\mathrm{i},t-1} + \Delta t \cdot \left(\frac{-5.18 \cdot 10^{-3} \cdot (\mathrm{I}_T \text{ or } \mathrm{I}_L)}{(\text{area} \cdot \text{depth})} - 1.0 \cdot [\mathrm{Ca}^{2+}]_{\mathrm{i},t-1}\right).$$

Perigeniculate Nucleus model cell (PGN)

For this cell we take all equations after Destexhe 1994.

General equation describing the change in intracellular potential in PGN model cell:

$$C_m \frac{\mathrm{d}V}{\mathrm{d}t} = -I_{\mathrm{inj}} - I_{\mathrm{T}} - I_{\mathrm{K[Ca]}} - I_{\mathrm{CAN}} - I_{\mathrm{Na}} - I_{\mathrm{K}} - I_{\mathrm{leak}}$$

Low threshold Ca^{2+} current I_T

The current density is given by:

$I_{\mathrm{T}} = I_{\mathrm{T,max}}(V - E_{\mathrm{Ca}})m^2 h,$

where E_{Ca} is the calcium reversal potential, see below. We use the following activation and inactivation kinetics:

$$\tau_m = 0.44 + 0.15\left[\exp\left(\frac{V+27}{10}\right) + \exp\left(-\frac{V+102}{15}\right)\right]^{-1},$$

$$m_\infty = \left[1 + \exp\left(-\frac{V+52}{7.4}\right)\right]^{-1},$$

$$\tau_h = 22.7 + 0.27\left[\exp\left(\frac{V+48}{4}\right) + \exp\left(-\frac{V+407}{50}\right)\right]^{-1},$$

$$h_\infty = \left[1 + \exp\left(\frac{V+80}{5}\right)\right]^{-1}.$$

Ca^{2+} dependent K^+ current $I_{K[Ca]}$

$I_{\mathrm{K[Ca]}} = I_{\mathrm{K[Ca],max}}(V - E_{\mathrm{K}})m^2$,

where $E_{\mathrm{K}} = -95\mathrm{mV}$. The activation variable m is calculated from

$$\frac{\mathrm{d}m}{\mathrm{d}t} = -\frac{1}{\tau_m([\mathrm{Ca}^{2+}]_{\mathrm{i}})}(m - m_\infty([\mathrm{Ca}^{2+}]_{\mathrm{i}}))$$

with the parameters depending on the intracellular calcium concentration:

$$m_\infty([\mathrm{Ca}^{2+}]_{\mathrm{i}}) = \frac{\alpha \cdot [\mathrm{Ca}^{2+}]_{\mathrm{i}}^n}{\alpha \cdot [\mathrm{Ca}^{2+}]_{\mathrm{i}}^n + \beta},$$

$$\tau_m([\mathrm{Ca}^{2+}]_{\mathrm{i}}) = \frac{1}{\alpha \cdot [\mathrm{Ca}^{2+}]_{\mathrm{i}}^n + \beta},$$

where $\alpha = 48\mathrm{ms}^{-1}\mathrm{mM}^{-2}$, $\beta = 0.03\mathrm{ms}^{-1}$ and the exponent n is equal to 2.

Ca^{2+} dependent non-specific cation current I_{CAN}

$I_{\mathrm{CAN}} = I_{\mathrm{CAN}} m^2(V - E_{\mathrm{CAN}})$,

where $E_{\mathrm{CAN}} = -20\mathrm{mV}$. Activation variable's (m) kinetics given by:

$$\tau_m = \frac{1}{\alpha \cdot [\mathrm{Ca}^{2+}]_{\mathrm{i}}^n + \beta},$$

$$m_\infty = \frac{\alpha \cdot [\mathrm{Ca}^{2+}]_{\mathrm{i}}^n}{\alpha \cdot [\mathrm{Ca}^{2+}]_{\mathrm{i}}^n + \beta},$$

with $\alpha = 20\mathrm{ms}^{-1}\mathrm{mM}^{-2}$, $\beta = 0.002\mathrm{ms}^{-1}$ and $n = 2$.

Fast Na^+ current

The Na^+ was modeled using Hodgkin-Huxley formalism (Hodgkin and Huxley, 1952). The current density is given by:

$I_{\mathrm{Na}} = I_{\mathrm{Na,max}}(V - E_{\mathrm{Na}})m^3h$,

Rate constants of the activation variable m:

$$\alpha = 0.1(V + 40)\left[1 - \exp\left(\frac{-V - 40}{10}\right)\right]^{-1},$$

$$\beta = 4 \cdot \exp\left(\frac{-V - 65}{18}\right).$$

Rate constants of the inactivation variable h:

$$\alpha = 0.07\exp\left(\frac{-V - 65}{20}\right),$$

$$\beta = \left[\exp\left(\frac{-V - 35}{10}\right) + 1\right]^{-1}.$$

Fast K^+ current

The K^+ was modeled using Hodgkin-Huxley formalism (Hodgkin and Huxley, 1952). The current density is given by:

$I_{\mathrm{K}} = I_{\mathrm{K,max}}(V - E_{\mathrm{K}})m^4$,

Rate constants of the activation variable m:

$$\alpha = 0.01(-V - 55)\left[\exp\left(\frac{-V - 55}{10}\right) - 1\right]^{-1},$$

$$\beta = 0.125 \cdot \exp\left(\frac{-V - 65}{80}\right).$$

Non-specific leak current I_{leak}

$I_{\mathrm{leak}} = I_{\mathrm{leak,max}}(V - E_L)$

Effective reversal potential $E_L = -78\mathrm{mV}$.

Ca^{2+} concentration and buffering

Ca^{2+} influx:

$$\frac{\mathrm{d}[\mathrm{Ca}^{2+}]_{\mathrm{i}}}{\mathrm{d}t} = -\frac{K}{2Fd}I_{\mathrm{T}},$$

where $d = 1\mu\mathrm{m}$ is the depth of the shell beneath the membrane and $k = 0.1$ is a factor resulting from conversion of units (for I_{T} in $\mu\mathrm{A/cm}^2$).

Ca^{2+} pump contribution:

$$\frac{\mathrm{d}[\mathrm{Ca}^{2+}]_{\mathrm{i}}}{\mathrm{d}t} = -\frac{K_{\mathrm{T}} \cdot [\mathrm{Ca}^{2+}]_{\mathrm{i}}}{[\mathrm{Ca}^{2+}]_{\mathrm{i}} + K_{\mathrm{d}}},$$

with $K_{\mathrm{T}} = 10^{-4}\mathrm{mM/ms}$ and $K_{\mathrm{d}} = 10^{-4}\mathrm{mM}$.

Ca^{2+} buffering term:

$$\frac{\mathrm{d}[\mathrm{Ca}^{2+}]_{\mathrm{i}}}{\mathrm{d}t} = \frac{[\mathrm{Ca}^{2+}]_{\mathrm{i}} - [\mathrm{Ca}^{2+}]_{\mathrm{i},\infty}}{\tau_r}$$

where $\tau_r = 500\mathrm{ms}$ is calcium removal rate constant, and $[\mathrm{Ca}^{2+}]_{\mathrm{i},\infty} = 2.4 \cdot 10^{-4}\mathrm{mM}$.

Ca^{2+} reversal potential:

$$E_{\mathrm{Ca}} = k'\frac{RT}{2F}\log\frac{[\mathrm{Ca}^{2+}]_{\mathrm{o}}}{[\mathrm{Ca}^{2+}]_{\mathrm{i}}},$$

$k' = 1000$ being a conversion factor for E_{Ca} in mV.

Feed-forward interneuron model cell (Int)

Currents used in the Int model cell come from TC and PGN models as described above. General equation describing the change in intracellular potential in Int model cell:

$$C_m \frac{\mathrm{d}V}{\mathrm{d}t} = -I_{\mathrm{inj}} - I_{\mathrm{T}} - I_{\mathrm{K[Ca]}} - I_{\mathrm{L}} - I_{\mathrm{CAN}} - I_{\mathrm{h}} - I_{\mathrm{C}} - I_{\mathrm{Na}} - I_{\mathrm{K}}.$$

Calcium dynamics followed the TC cell model.

Cortex model cell (Cx)

Cortex cell was modeled using Hodgkin-Huxley formalism and currents (Hodgkin and Huxley, 1952) as described in PGN model cell for the I_{Na} and I_{K} currents.
